# Endoscopic hemostasis with endoscopic mucosal resection and multiple synchronous early gastric cancers: a case report

**DOI:** 10.1186/1752-1947-6-268

**Published:** 2012-08-31

**Authors:** Shintaro Fujihara, Hirohito Mori, Noriko Nishiyama, Mitsuyoshi Kobayashi, Hideki Kobara, Tsutomu Masaki

**Affiliations:** 1 Department of Gastroenterology and Neurology, Kagawa University, Faculty of Medicine, 1750-1 Ikenobe, Miki-cho, Kita-gun, Kagawa Prefecture 761-0793, Japan

## Abstract

**Introduction:**

Endoscopic hemostasis for severe upper gastrointestinal bleeding due to tumors, such as gastrointestinal stromal tumors and malignant lymphoma, is temporarily effective. However, permanent hemostasis is difficult in many cases because of diffuse bleeding.

**Case presentation:**

A 60-year-old Japanese woman was admitted to our hospital with hematemesis. Endoscopy revealed multiple gastric polyps and fresh blood in her stomach. One of the gastric polyps, which was associated with oozing bleeding, was found near the anterior wall of the lower gastric body. We initially applied hemostatic forceps and argon plasma coagulation over the tumor surface, but the bleeding persisted. After endoscopic mucosal resection, exposed vessels were seen at the base of the mucosal resection site with oozing bleeding. Coagulation of the bleeding vessels using hemostatic forceps allowed successful completion of the hemostatic procedure. Our patient also had eight synchronous gastric cancer lesions. Histological examination of the resected specimens showed various types of cancer.

**Conclusion:**

This is a case report of gastric cancer associated with eight gastric cancer lesions, confirmed by histology, in which hemostasis was achieved through endoscopy.

## Introduction

Endoscopic hemostasis for severe upper gastrointestinal (GI) bleeding due to tumors, such as GI stromal tumors and malignant lymphoma, is temporarily effective [1]. However, permanent hemostasis is difficult in many cases because of diffuse bleeding [2]. We here report a case of endoscopic hemostasis with endoscopic mucosal resection (EMR) as a rescue therapy for bleeding of a malignant GI tumor after failed conventional therapy. This patient also had multiple synchronous gastric cancers.

## Case presentation

A 60-year-old Japanese woman was admitted to our hospital with hematemesis. She had no history of GI hemorrhage, peptic ulcer disease, use of nonsteroidal anti-inflammatory drugs, excessive alcohol ingestion, or chronic liver disease. She had no other medical problems and was taking no medications.

On presentation, her abdomen was soft, nontender, and nondistended with normal bowel sounds. Her hemoglobin level was 8.5g/dL. Contrast-enhanced computed tomography (CT) revealed multiple lesions in her stomach. Hematologic tests revealed a total peripheral white cell count of 14,400/mm^3^. Biochemical tests yielded elevated serum blood nitrogen (30.7mg/dL), decreased total protein (3.8g/dL), and decreased serum albumin (2.0mg/dL).

**Figure 1:**
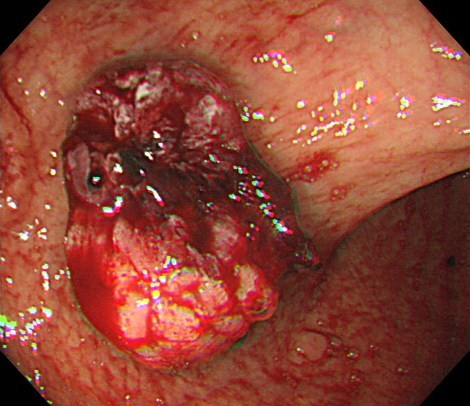
Oozing bleeding was observed from the gastric cancer lesion after argon plasma coagulation.

Endoscopy revealed multiple gastric polyps and fresh blood in her stomach. One of the gastric polyps, which was associated with oozing bleeding, was found near the anterior wall of the lower gastric body. Hemostatic forceps (Coagrasper, FD-410LR; Olympus, Tokyo, Japan) were initially applied over the tumor surface, but because the bleeding persisted, argon plasma coagulation (APC) was performed around the bleeding site. Mucosal oozing continued after failed APC and the application of the hemostatic forceps (Figure 1).

Bleeding of this polyp lesion persisted, and we did not determine whether it was a benign or a malignant tumor (in particular, hyperplastic poly or early gastric cancer). We considered that the bleeding point was not only on the surface of this polyp, but also within the deep mucosal layer.

**Figure 2:**
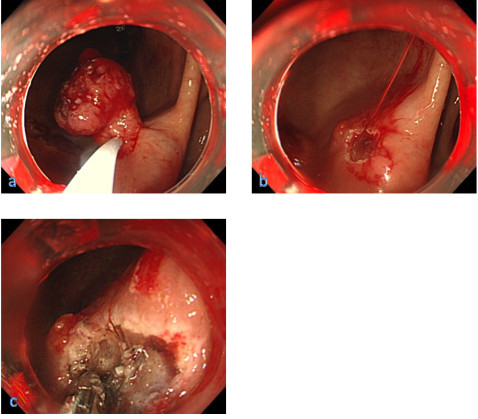
**Endoscopic views before and after endoscopic mucosal resection.** (**a**) Exposed blood vessels in the gastric cancer lesion treated by endoscopic mucosal resection. (**b**) After endoscopic mucosal resection, exposed vessels were seen at the base of the mucosal resection site with spurting bleeding. (**c**) Endoscopic hemostasis was achieved using hemostatic forceps.

**Figure 3:**
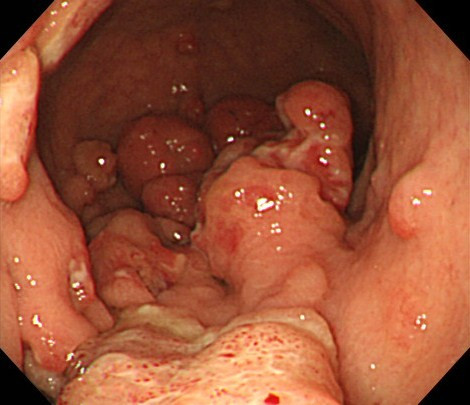
The following day, endoscopy revealed multiple lesions in the stomach.

**Figure 4:**
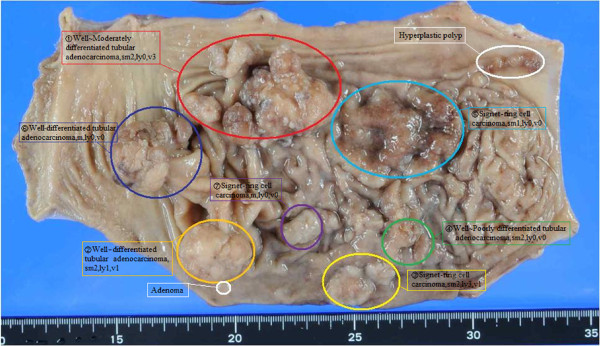
Gastrectomy specimen showing multiple early gastric cancers in the body of the stomach.

**Figure 5:**
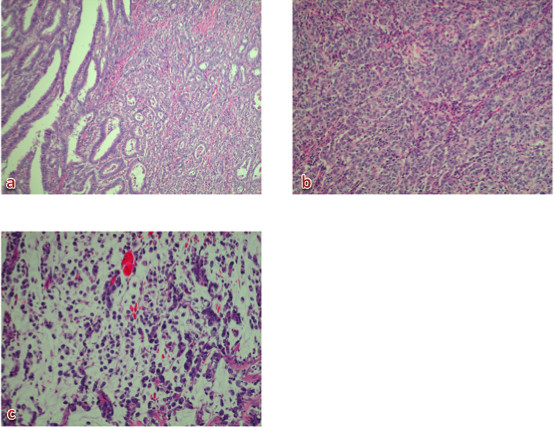
**Histological examination showed various types of cancer: well-differentiated, moderately-differentiated, and poorly-differentiated adenocarcinoma and signet-ring cell carcinoma that had developed independently in the mucosal and submucosal layers of the resected specimen.** (**a**) No. 1 revealed a well-differentiated adenocarcinoma with a moderately-differentiated adenocarcinoma. (**b**) No. 4 revealed a well-differentiated adenocarcinoma with a moderately-differentiated adenocarcinoma. (**c**) No. 5 revealed a signet-ring cell carcinoma.

We decided to perform EMR to detect the deep vessels. Immediately after injecting a 0.4% sodium hyaluronate solution, EMR was performed (Figure 2a). After EMR, exposed vessels were detected at the base of the mucosal resection site, with spurting bleeding (Figure 2b). Coagulation of the bleeding vessels using an ERBE VIO® 200 generator (ERBE Elektromedizin, Tübingen, Germany) and hemostatic forceps allowed us to successfully complete the hemostatic procedure (Figure 2c). The bleeding stopped within two days. Our patient’s hemoglobin level subsequently stabilized at 10.2g/dL without further transfusion. The following day, upper GI endoscopy showed multiple gastric cancer lesions (Figure 3). There was no further bleeding. Subsequent examination of specimens from EMR revealed well-differentiated adenocarcinoma. The lesion was removed *en bloc*. The resected tumor was 30mm × 25mm, and histological examination revealed well-differentiated tubular adenocarcinoma limited to the submucosal layer. Biopsy specimens that were obtained from other lesions revealed well-differentiated adenocarcinoma and signet-ring cell carcinoma. There was no evidence of hepatic metastasis, lung metastasis, or peritoneal metastasis based on the CT and positron emission tomography findings. Total gastrectomy with a radical lymph node dissection was performed with a preoperative diagnosis of multiple gastric cancers. This patient had eight synchronous gastric cancer lesions (Figure 4). Histological examination showed various types of cancer (Figure 5a-c): well-differentiated, moderately-differentiated, and poorly-differentiated adenocarcinoma and signet-ring cell carcinoma that had developed independently in the mucosal and submucosal layers of the resected specimen. Our patient’s symptoms resolved after surgery, and she remained asymptomatic at the follow-up one-and-a-half years later.

## Discussion

This is a report of gastric cancer associated with eight gastric cancer lesions, proven on histopathology, in which hemostasis was obtained by endoscopic treatment. Malignant tumors of the stomach and duodenum frequently cause chronic GI blood loss and anemia, but acute upper GI bleeding is uncommon (2% to 4% of episodes of acute upper GI bleeding). The mortality rate for patients with gastric carcinoma presenting with acute hemorrhage is extremely high [3-5].

The reported median survival for patients with acute tumor bleeding ranges from 39 days to 12 weeks [3,4]. Endoscopic hemostasis for severe upper GI bleeding due to tumors, such as GI stromal tumors and malignant lymphoma, is temporarily effective [1]. APC is particularly useful for identification of the vessels responsible for the bleeding [6,7]. However, permanent hemostasis is difficult in many cases because of diffuse bleeding. Early consultation with a surgeon is warranted when hemostasis is difficult to achieve. Hemostatic forceps may be an effective and safe alternative approach for active GI bleeding of various origins. The incidence of multiple synchronous gastric cancers (MSGC) is thought to be about 4% to 15% in patients with gastric cancer [8,9]. Our patient had seven synchronous gastric cancer lesions. Patients with two or three cancer lesions are frequently seen; however, there are few reports describing patients with four or more multiple gastric cancer lesions. Cases of MSGC with seven or more lesions are very rare [10]. A review of the literature revealed that many patients with MSGC had accessory lesions that were overlooked preoperatively and detected after surgery by histological examination [11,12]. Sixteen patients (11%) had synchronous multiple early gastric cancer lesions within one year of the initial EMR. About half of the multiple lesions were located in the same third of the stomach as the primary lesion, and most lesions were similar in macroscopic type to the primary lesions. Most multiple lesions were of the differentiated type.

## Conclusions

In our opinion, EMR might be suitable as a bridging therapy for bleeding tumors after other failed interventions, but not as the primary method of treatment. In addition, we report a rare case of multiple synchronous early gastric carcinomas with eight lesions.

## Consent

Written informed consent was obtained from the patient for publication of this manuscript and accompanying images. A copy of the written consent is available for review by the Editor-in-Chief of this journal.

## Competing interests

The authors declare that they have no competing interests.

## Authors’ contributions

SF, HM, NN, MK and HK made substantial contributions to the acquisition and interpretation of data. TM gave final approval of the version to be published. All authors read and approved the final manuscript.

## Authors’ information

All authors are specialized in the diagnosis and treatment of all diseases in both the upper and lower GI tract. Research projects of our team include clinical and basic research for advanced endoscopic therapy, especially endoscopic submucosal dissection and natural orifice transluminal endoscopic surgery.
